# A mechanical rotation chair provides superior diagnostics of benign paroxysmal positional vertigo amongst elderly

**DOI:** 10.3389/fneur.2026.1873559

**Published:** 2026-07-15

**Authors:** Dan Dupont Hougaard, Bertram Vorm, Regitze Gyldenholm Skals, Jesper Holm, Jens Faunoe Thrane, Michael Lüscher

**Affiliations:** 1 Balance & Dizziness Center, Department of Otorhinolaryngology, Head & Neck Surgery and Audiology, Aalborg University Hospital, Aalborg, Denmark; 2 Department of Clinical Medicine, Aalborg University, Aalborg, Denmark; 3 Research Data and Biostatistics, Aalborg University Hospital, Aalborg, Denmark; 4 Aarhus ENT Practice, Luscher & Thrane, Aarhus, Denmark

**Keywords:** benign paroxysmal positional vertigo, BPPV, diagnostics, mechanical rotation chair, Rotundum rotary chair, vertigo

## Abstract

**Background:**

Benign Paroxysmal Positional Vertigo (BPPV) is the most common cause of vertigo and occurs frequently among elderly individuals, who are at increased risk of falls, impaired mobility, and reduced quality of life. Traditional bedside (TB) diagnostics rely on accurate patient positioning and cooperation, which may be challenging in older populations. Mechanical rotation chairs (MRCs) have been introduced to improve diagnostic precision, but evidence regarding their superiority in elderly patients remains limited.

**Objective:**

To compare the diagnostic accuracy of BPPV between MRC- and TB diagnostics in an elderly population.

**Methods:**

Prospective, randomized crossover study with 201 participants aged 60 years or above. Participants underwent both TB- and MRC diagnostics with bilateral Dix-Hallpike- and Supine Roll Tests. The order of diagnostic modality was randomized. Agreement between modalities was assessed using percentage agreement and Cohen's kappa. Sensitivity, specificity, positive predictive value (PPV), and negative predictive value (NPV) of TB diagnostics were calculated using MRC diagnostics as the reference standard. Secondary analyses examined diagnostic performance in posterior versus non-posterior BPPV and the effect of reduced patient cooperation. Tertiary analyses examined optimal positioning with MRC Supine Roll Testing.

**Results:**

BPPV was diagnosed in 61 participants (30.3%) with TB diagnostics and 68 participants (33.8%) with MRC diagnostics. Overall agreement between modalities was 89.6% with a Cohen's kappa of 0.76, indicating substantial to near-perfect agreement. Compared with MRC diagnostics, TB diagnostics demonstrated a sensitivity of 79%, specificity of 95%, PPV of 89%, and NPV of 90%. Sensitivity for non-posterior BPPV was lower (63%). Diagnostic performance declined in participants with impaired cooperation, where sensitivity decreased to 60%. MRC diagnostics identified more non-posterior and cupulolithiasis cases than TB diagnostics. A 60-degree backward Supine Roll Test position showed higher detection rates of lateral BPPV than a 90-degree position, although differences were not statistically significant.

**Conclusion:**

MRC diagnostics provide superior diagnostic sensitivity for BPPV in elderly patients, particularly for non-posterior subtypes and in cases with limited patient cooperation. TB diagnostics remain an effective first-line diagnostic tool. However, MRC diagnostics should be considered when bedside findings are inconclusive or when complex BPPV is suspected.

**Clinical trial registration:**

ClinicalTrials.gov, identifier: NCT04147156.

## 1 Introduction

Benign Paroxysmal Positional Vertigo (BPPV) is by far the single most frequent cause of vertigo. As many as 17–42% of patients complaining of vertigo might be due to BPPV (1). The disease has a 1-year prevalence of 1.6%, a 1-year incidence of 0.6% and a lifetime prevalence of 2.4% (2). Several epidemiological studies have shown a female preponderance of approximately two to one (2:1 female/male ratio) (3). With age, the cumulative lifetime incidence rises to approximately 10% at 80 years of age and the incidence peaks in the fifth-, sixth-, and seventh decades (2).

A typical BPPV case history includes symptoms of recurrent, brief episodes of vertigo (spinning sensation) or dizziness, consistently triggered by specific changes in head position relative to gravity (4). Two distinct subtypes describe the condition: (1) otoliths that adhere to the cupula which is termed *cupulolithiasis* (CUP) (this concept was introduced by Schuknecht in 1969), and (2) free floating otoliths in one or several of the semicircular canals (SCCs) which is termed *canalolithiasis* (CAN) (this concept was introduced by Hall et al. in 1979). Also, BPPV can be subdivided into primary (idiopathic) or secondary BPPV, which refers to BPPV as a sequela to previous or existing disease within the inner ear, e.g. Meniere's disease, vestibular neuritis, labyrinthitis, and/or head trauma.

Diagnostics of BPPV is predominantly done with the aid of predefined diagnostic positional tests on an examination bed. For testing of the vertical SCCs, a left and right Dix Hallpike (DH) test is recommended and for testing of the horizontal SCCs, a left and right supine roll test (SRT) is recommended (4, 5). Given its high prevalence, BPPV is frequently encountered and managed by a broad spectrum of healthcare professionals across primary, secondary, and tertiary care sectors.

Several well-defined diagnostic criteria exist. Among these, the criteria set forth by the Bárány Society and the American Association of Otorhinolaryngology, Head & Neck Surgery (AAO-HNS) are the most widely used (4, 5). However, these diagnostic criteria, which were defined in 2015 and 2017, respectively, may no longer accurately reflect contemporary evidence and clinical practice.

As a prerequisite, both criteria state that the patient should present with a characteristic BPPV case history (refer to previous description in the beginning of the introduction). They also agree that the patient should have objectifiable BPPV-characteristic positional nystagmus with either the DH test and/or the SRT. However, only the AAO-HNS criteria, which are based on current evidence, require and state that the patient must also experience a subjective feeling of vertigo concomitant to the observation of their positional nystagmus.

To some extent, BPPV diagnostics require both a skilled and trained health care professional to position the patient correctly and to identify, observe, and describe any positional nystagmus encountered. However, even experienced and highly trained health care professionals are not continuously and repeatedly able to position the patient accurately during neither traditional bedside (TB) diagnostics nor during traditional bedside treatment maneuvers on an examination bed. One study has found a variation of the intended head position with the Epley maneuver as high as 65 degrees with even experienced health care professionals (6).

Also, sufficient patient cooperation is very important with TB diagnostics on an examination bed. If the patient cannot fully cooperate, then optimal positioning will be compromised. One study found significantly reduced sensitivity, specificity and negative predictive value if patients had impaired but acceptable degree of cooperation during diagnostic positioning (7).

As previously stated, elderly patients are especially at risk of getting BPPV. BPPV-related sequelae in this subpopulation of patients are also worth mentioning. Elderly patients with BPPV are at greater risk of falls, depression, and impaired daily function (8). Low degree of patient cooperation might especially be an issue among elderly patients with severely reduced mobility as well as various neck issues. Also, because of vestibular agnosia, symptoms among elderly patients with BPPV may indeed not be so characteristic as with the remaining part of the BPPV population (9).

Despite high success rates with traditional BPPV treatment on an examination bed, not all types of BPPV are curable by traditional means. In general, single canal BPPV is easier to treat and has a lower risk of recurrence(s), compared to multiple canal BPPV. A systematic review from 2023 stated that failure of treatment using traditional bedside repositioning maneuvers was found in 18.4% of cases (10).

During the last couple of decades, several mechanical rotation chairs (MRCs) have been developed to improve BPPV diagnostics and -treatments. In 1996, the American Epley Omniax^®^ System chair was introduced by Dr John Epley. In 2003, the French Thomas Richard-Vitton (TRV^®^) reposition chair was developed, and in the late 2010s the Rotundum^®^ rotary chair was developed in Switzerland. Overall features of MRCs include very precise and reproducible positioning of patients with 360-degree rotations in all three planes (yaw-, roll-, and pitch axes) during positional testing as well as during subsequent repositioning maneuvers. Additionally, VNG goggles are used to optimize visualization of eye movements (e.g. automatic pupil tracking, nystagmus slow phase velocity measurements, eye movement recordings and analysis of eye movements). Diagnostic studies have shown that MRCs enable superior BPPV diagnostics with all other BPPV subtypes and -locations other than posterior CAN BPPV (7, 11). Moreover, previous studies have shown that MRCs offer very successful treatment rates from 92–94% even with complicated BPPV cases including multiple canal BPPV, CUP subtype BPPV, as well as refractory BPPV cases requiring up to ten treatments by means of an MRC (12–14). Current recommendations include the use of MRCs for *BPPV diagnostics* in case of reduced cooperation during traditional diagnostics on an examination bed, a typical BPPV case history with negative (normal) positional testing, and non-posterior BPPV cases where exact classification in terms of localization, subtype, and laterality proves challenging (7, 11). Current recommendations for the use of *MRC BPPV treatment* include reduced patient cooperation during canalith reposition maneuvers (CRMs) on an examination bed, atypical BPPV locations, multiple canal BPPV, CUP subtype BPPV or otherwise CRM refractory BPPV cases (12–15).

A fairly new, Swiss made, MRC has not yet been thoroughly studied. Current literature has shown that this MRC can be used for: (1) safe and efficient treatment of BPPV, (2) out-of-hospital BPPV diagnostics and -treatments, and (3) very refractory BPPV cases requiring more than ten MRC treatments (16–18). Unique features with this MRC include portability (by means of wheels and folding capabilities) as well as stepless 360-degree rotations in all three axes with exact (one-degree) positioning for diagnostics and treatments. This MRC does not have a cushion or a similar feature that ensures a 30-degree forward flexion of the head (and thereby vertical alignment of the lateral SCCs during SRTs) like other MRCs. Therefore, the optimal positioning of the patient for SRT testing with this MRC is not known.

This study was designed to examine BPPV diagnostic accuracy with one type of MRC compared to TB diagnostics on an examination bed in a population of elderly patients. Therefore, the primary endpoint was to investigate the overall agreement between BPPV diagnostics performed with an MRC and TB diagnostics on an examination bed in a population of elderly patients. The first secondary objective of this study was to examine if sensitivity and specificity of TB diagnostics differed when diagnosing uncomplicated BPPV cases and complicated BPPV cases (defined at the end of this section). A second secondary objective was to determine whether poor patient cooperation during TB diagnostics affected the level of agreement, sensitivity, and/or specificity. The third endpoint of this study included evaluation of the optimal position with this MRC for SRT testing (60-degree or 90-degree backwards starting position in supine).

Uncomplicated BPPV cases in this study included patients who consulted an otorhinolaryngologist at an Ear-, Nose-, and Throat Practice (referred to as an ENT Practice in the remainder of this article) where patients in Denmark may undergo diagnostics and treatment by an ENT specialist without prior referral. Complicated BPPV cases included patients referred to a highly specialized University Hospital based Dizziness Center (tertiary sector). Uncomplicated BPPV cases therefore refer to patients from the primary sector [self-referrals or referrals from general practitioners (GPs)] and complicated BPPV cases refer to patients referred from the secondary sector [referrals from an ENT Practice] to a highly specialized unit.

## 2 Methods

### 2.1 Study design

This study applied an open-labeled randomized diagnostic crossover design adhering to the Consolidated Standards of Reporting Trials (CONSORT) guidelines. The trial was approved by the North Denmark Region Committee on Health Research Ethics (approval number 2024-0024). The studies were conducted in accordance with the local legislation and institutional requirements. The participants provided their written informed consent to participate in this study. Written informed consent was obtained from the individual(s) for the publication of any identifiable images or data included in this article. Prior to study initiation, the study was registered at the following clinical trial registration site: ClinicalTrials.gov with identifier: NCT04147156.

All participants underwent the recommended diagnostic procedures with both the DH test and the SRT bilaterally. Both diagnostic procedures were done by TB diagnostics and also by MRC diagnostics (4, 5). For each participant, the initial test modality was chosen on the day of examination by randomization with an allocation ratio of 1:1. To prevent fatiguability of positional nystagmus and accompanying positional vertigo, the two diagnostic tests were separated by a minimum of 15 min (end of initial examination with the first test modality until the initiation of the following examination with the second test modality) (7). Prior to inclusion, all examiners underwent clinical training in the use of both diagnostic modalities and were supervised by two different neurotologists throughout the inclusion period. Due to the nature of the study, blinding of participants and examiners was not possible.

### 2.2 Participants and setting

Participants were included during a 13-month period between October 2024 and November 2025 at two different centers: (1) A tertiary University Hospital-based outpatient clinic (Balance & Dizziness Clinic, Department of Otorhinolaryngology, Head & Neck Surgery and Audiology, Aalborg University Hospital, Aalborg, Denmark) or (2) a secondary ENT Practice (Aarhus ENT Practice, Luscher & Thrane, Aarhus, Denmark). All participants had to present with a typical BPPV case history (4, 5). With the University hospital-based clinic, potential study participants were referred by general practitioners in the North Denmark Region and ENTs in the North and Central Denmark Regions. With the ENT Practice, potential study participants were self-referrals or referrals from GPs.

Upon referral, study eligibility was decided by the individual examiner according to the predefined study in- and exclusion criteria. Inclusion criteria included age at or above 60 years of age, medical history compatible with BPPV [symptoms of recurrent, brief episodes of vertigo or dizziness, consistently triggered by specific changes in head position relative to gravity (4)], and the ability to read and understand Danish. Exclusion criteria included physical or mental inability to cooperate during the positional tests with both test modalities (e.g. claustrophobia or cervical spine immobility), known cerebral aneurysm, recent cerebrovascular events (within 1 month), bodyweight above 150 kilograms, and cognitive limitations.

Prior to enrollment, all participants were given oral and written information about the study. Written consent was obtained before initial randomization that determined which test modality the participant should undergo initially.

### 2.3 Materials

Both TB- and MRC diagnostics were performed with the aid of video nystagmography goggles (VNG) (ICS Impulse^®^, Natus Sensory(*c*), Middleton, WI, US) with accompanying software [OTOsuite vestibular^®^ (version 4.31.1690), Natus Medical Incorporated, Middleton, WI, US]. This enabled optimization of eye monitoring by enlargement of the eyes, recording- and quantification of eye movements. The MRC used with this study was the Rotundum rotary chair^®^ (balcare GmbH©, Küsnacht, Switzerland). Upon medical history taking and clinical examination of the participants, a diagnosis of BPPV was either placed or ruled out. Prerequisites for placing a diagnosis of BPPV included the combination of (1) a classical BPPV case history and (2) objective findings of BBPV characteristic positional nystagmus according to the criteria set forth by Von Brevern et al. (4). Identical criteria were used with both MRC- and TB diagnostics. If BPPV-characteristic positional nystagmus was observed, it was classified according to the criteria listed in Table 1.

**Table 1:** Classification of BPPV localization and–subtype.

BPPV localization	BPPV subtype	
	Canalolithiasis	Cupulolithiasis
Anterior SCC (Contralateral DH Test)	Downbeat vertical nystagmus ± rotational component beating away from the affected side with a duration of less than 1 min.	Downbeat vertical nystagmus ± rotational component beating away from the affected side with a duration of more than 1 min.
Lateral SCC (SRT)	Bilateral geotropic horizontal nystagmus with a duration of less than 1 min.	Bilateral apogeotropic horizontal nystagmus with a duration of more than 1 min.
Posterior SCC (DH test)	Upbeat nystagmus (with latency) with a rotational component beating toward the affected side with a duration of less than 1 min.	Upbeat nystagmus (without any latency) with a rotational component beating toward the affected side with a duration of more than 1 min.

The BPPV classifications were based on the characteristics of the objectifiable positional nystagmus observed during the recommended standard test positions (4). SCC, Semicircular Canal. DH, Dix-Hallpike. SRT, Supine Roll Test.

### 2.4 Interventions

Prior to positional testing, all participants underwent clinical examination with evaluation of spontaneous nystagmus (with and without fixation), gaze-evoked nystagmus, and vestibular-ocular reflex (VOR) testing with and without suppression.

**Figure 1:**
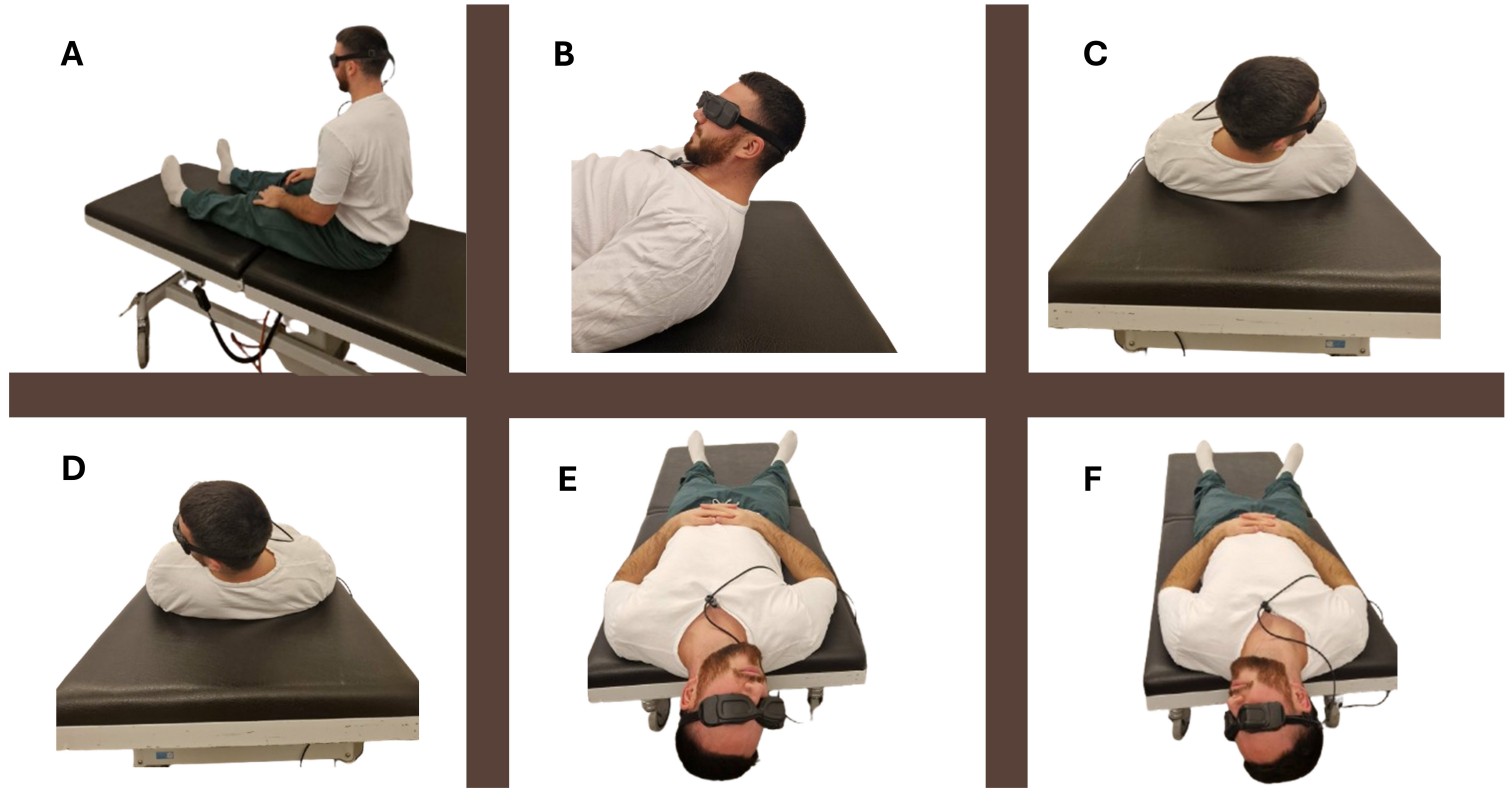
Traditional bedside BPPV diagnostics on an examination bed. **(A)** Participant in the upright position with VNG goggles in place. **(B)** Supine position with the head flexed 30 degrees above horizontal. **(C)** Right supine roll test with the head rotated 90 degrees to the right. **(D)** Left supine roll test with the head rotated 90 degrees to the left. **(E)** Right Dix-Hallpike test position. **(F)** Left Dix-Hallpike position. Please note that the examiner has been removed from the images to improve the visualization of the individual test positions.

**Figure 2:**
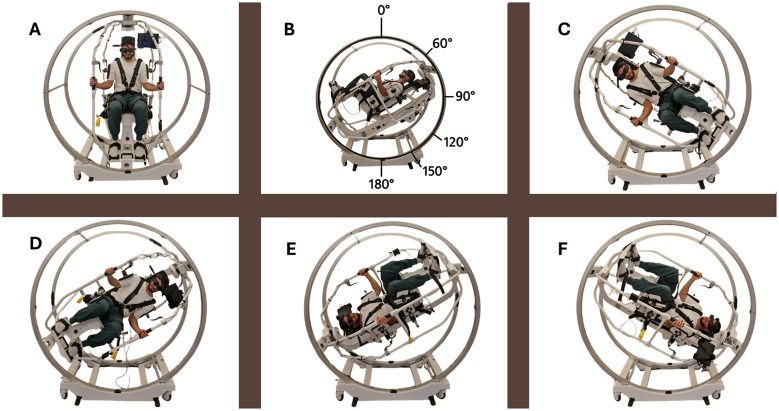
Type A diagnostics with the mechanical rotation chair. **(A)** Participant in the upright position with VNG goggles in place (vision denied). **(B)** Supine position with the participant positioned 60 degrees backwards in the roll plane. **(C)** Right supine roll test with the participant rotated 90 degrees to the right in the pitch plane. **(D)** Left supine roll test with the participant rotated 90 degrees to the left in the pitch plane. **(E)** Right Dix-Hallpike test position. **(F)** Left Dix-Hallpike test position.

**Figure 3:**
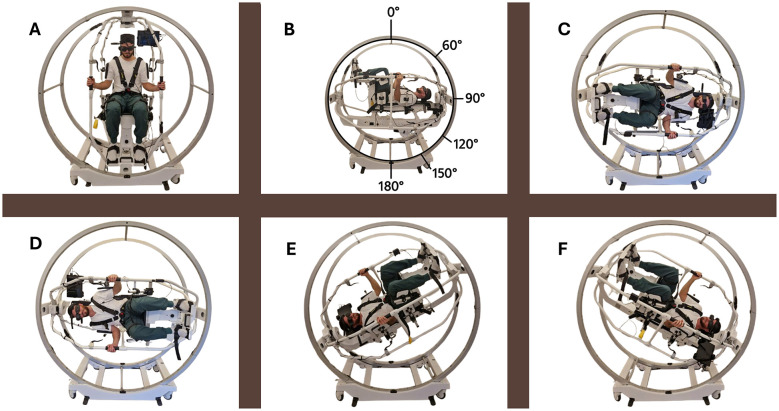
Type B diagnostics with the mechanical rotation chair. **(A)** Participant in the upright position with VNG goggles in place (vision denied). **(B)** Supine position with the participant positioned 90 degrees backwards in the roll plane. **(C)** Right supine roll test with the participant rotated 90 degrees to the right in the pitch plane. **(D)** Left supine roll test with the participant rotated 90 degrees to the left in the pitch plane. **(E)** Right Dix-Hallpike test position. **(F)** Left Dix-Hallpike test position.

Both TB- and MRC diagnostics included testing of the anterior-, lateral- and posterior SCCs. The same order of testing was used with both test modalities (supine position, SRT right and left, DH test right and left). Individual test positions are demonstrated in Figures 1–3.

With TB diagnostics, participants were placed in the supine position with the head at a 30-degree angle above the horizontal plane for 30 s. This was followed by a right SRT with the head turned 90 degrees to the right (with the head still being at a 30-degree angle above the horizontal plane). This position was held for 60 s. Then a left SRT followed with a head rotation 90 degrees to the left (with the head still being at a 30-degree angle above the horizontal plane). Participants were then placed upright, and a right DH test was performed. With the participant upright, the participant's head was turned 45 degrees to the right before moving the participant backwards to the final test position with the head 30 degrees below the horizontal plane. This position was held for 60 s. The participant was then again placed upright, and same procedure was done on the left side with the head turned 45 degrees to the left. In all test positions, the examiner observed and registered if any positional nystagmus and vertigo was encountered. The examiner also evaluated the degree of participant cooperation during the TB diagnostic procedures.

With MRC testing, the same order of positional testing was used. However, SRT was done in two separate ways to evaluate the tertiary endpoint dealing with angulation during SRT testing. Please refer to Figures 2, 3 for further details.

The video eye recordings could be reviewed by the examiner on-site and could therefore be used to support the overall BPPV diagnostics as well as subclassification according to laterality, location, and subtype. During TB diagnostics, examiners evaluated and recorded the degree of cooperation with each participant with these three categories: (1) cooperated well, (2) did not corporate well, but still acceptable, (3) was not able determine. *Please note that all diagnostic conclusions presented in this paper reflect the original decisions of the individual examiners directly following the diagnostic evaluations described*. All participants diagnosed with BPPV were offered and given treatment with the MRC used for this study. However, since this study is purely a diagnostic study, the outcome of treatments provided by the MRC are outside the aims and scope of this article and are therefore not included in the results.

### 2.5 Data collection

Data was collected on-site on the day of examination and inclusion. All data was uploaded to and managed on a secure server hosted by the University Hospital and the North Denmark Region. REDCap^®^ (Research Electronic Data Capture 15.5.15., 2025, Vanderbuilt University, Tennessee, USA) (19) was used for data storage. Furthermore, participants were asked to fill out the Danish version of the 25-item Dizziness Handicap Inventory questionnaire (DHI) on the day of examination (20).

### 2.6 Statistics

Background characteristics were presented as counts and frequencies for categorical variables and compared using a Chi Squared test. With small numbers (*n*), the Fishers Exact test was used for comparison. Continuous variables were presented as means and standard deviations (sd) and compared by an unpaired *T*-test. Non-normally distributed variables were presented by medians and range and compared by the Mann-Whitney U test.

Agreement on the BPPV diagnosis between MRC- and TB diagnostics was assessed using percent agreement and Cohen's Kappa to adjust for chance agreement. Furthermore, we measured sensitivity, specificity, negative predictive value (NPV), and positive predictive value (PPV) with TB diagnostics, by setting MRC diagnostics as the golden standard for BPPV diagnostics. We chose MRC diagnostics as the golden standard, because we were able to diagnose the highest amount of BPPV cases by this test modality. Analyses were performed for detection of BPPV overall, posterior BPPV, non-posterior BPPV, and for overall BPPV in different sub populations.

Diagnostics of lateral BPPV with the MRC were compared between two different test positions (60-degree vs. 90-degree Supine Roll Test position). Comparisons were performed by the Fishers Exact test.

All statistical analyses were performed using R version 4.2.2, and all tests were two-sided with a significance level of 0.05. All statistics were done by a certified biostatistician.

## 3 Results

**Figure 4:**
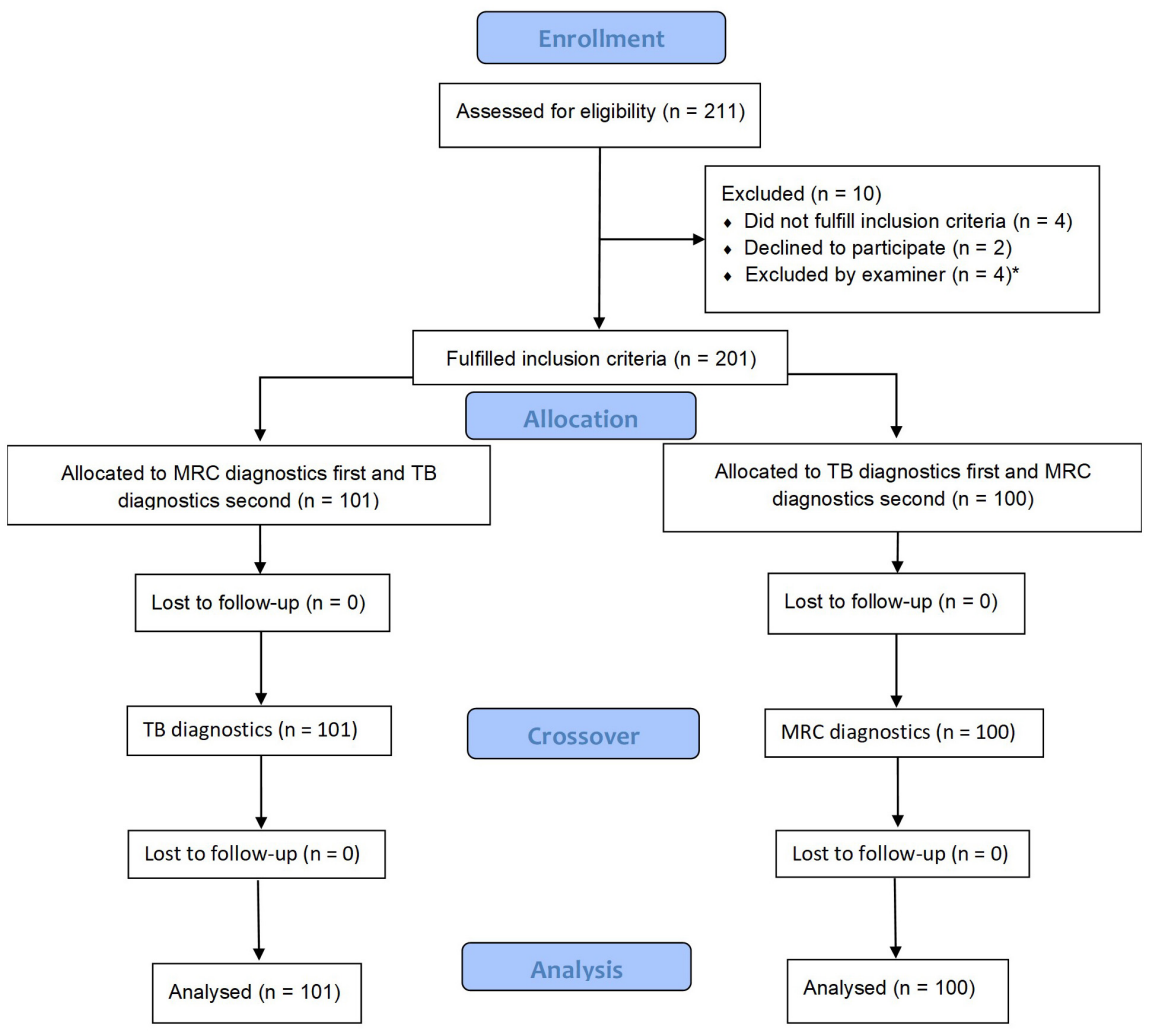
Trial profile. Diagram of included patients allocated to traditional bedside diagnostics (TB) and mechanical rotational chair diagnostics (MRC). *One participant was diagnosed with vestibular neuritis. Three participants were not able complete the entire examination (one because of excess vomiting, one was too weak to participate, and one was withdrawn due to cognitive limitations).

Following assessment of 211 potential study candidates, the study population ended up consisting of a total of 201 participants (please refer to Figure 4 for details on the trial profile). The proportion of males was 34.3%. The mean age was 74 years ranging from 60 to 97 years of age. Participants were referred from general practitioners (*n* = 140, 69.7%), private ENTs (*n* = 38, 18.9%), self-referrals (*n* = 17, (8.5%), or other hospital departments (*n* = 6, 3.0%). Overall, 61 (30.3%) and 68 (33.8%) participants were diagnosed with BPPV following TB- and MRC diagnostics, respectively. 53 (26.4%) and 60 (29.9%) were diagnosed with primary BPPV, and 8 (4.0%) and 8 (4.0%) were diagnosed with secondary BPPV following TB- and MRC diagnostics, respectively. When comparing the two study populations, significant differences were seen with the proportion of females, the proportion of participants diagnosed with BPPV, the number bilateral BPPV cases, the number of cup subtype BPPV, and also with the inter study site referral patterns. For details on baseline characteristics, please refer to Table 2.

**Table 2:** Background Characteristics.

Demographics	Total (*n* = 201)	Tertiary university hospital (*n* = 108)	ENT practice (*n* = 93)	*P*-value
Age				
Mean (SD)	74 (7.9)	74.2 (7.4)	73.8 (8.5)	0.692
Median [range]	74 [60, 97]	75 [60, 91]	74 [60, 97]	
Female, *n* (%)	132 (65.7)	63 (58.3)	69 (74.2)	0.027
Total DHI score, mean (SD)	39.6 (19.7)	^*^41.1 (20.3)	38 (19)	0.267
Duration of symptoms, days, median, (range), [IQR]	57, (0–4395), [25,125]	63, (0–4395), [29.5,139.5]	38, (5–2162), [22,120]	0.165
Referrals from GP, *n* (%)	140 (69.7)	70 (64.8)	70 (75.3)	
Referrals from an ENT Practice	38 (18.9)	32 (29.3)	6 (6.5)	
Referrals from a hospital department	6 (3.0)	6 (5.6)	0 (0.0)	
Self-referrals	17 (8.5)	0 (0.0)	17 (18.3)	< 0.001
Primary BPPV, *n* (%)				
TB	53 (26.4)	36 (33.3)	17 (18.3)	0.024
MRC	60 (29.9)	40 (37.0)	20 (21.5)	0.025
Secondary BPPV, *n* (%)				
TB	8 (4.0)	3 (2.8)	5 (5.4)	0.475^**^
MRC	8 (4.0)	5 (4.6)	3 (3.2)	0.727^**^
BPPV localization and -subtype				
BPPV overall, *n* (%)				
TB	61 (30.3)	39 (36.1)	22 (23.7)	0.078
MRC	68 (33.8)	45 (41.7)	23 (24.7)	0.017
BPPV right side, *n* (%)				
TB	31 (15.4)	22 (20.4)	9 (9.7)	0.058
MRC	41 (20.4)	27 (25.0)	14 (15.1)	0.117
BPPV left side, *n* (%)				
TB	26 (12.9)	14 (13.0)	12 (12.9)	1.000
MRC	22 (10.9)	13 (12.0)	9 (9.7)	0.758
Bilateral BPPV, *n* (%)				
TB	3 (1.5)	3 (2.8)	0 (0.0)	0.250^**^
MRC	4 (2.0)	4 (3.7)	0 (0.0)	0.125^**^
Mono-canal BPPV, *n* (%)				
TB	57 (28.4)	38 (35.2)	19 (10.4)	0.031
MRC	64 (31.8)	43 (39.8)	21 (22.6)	0.014
Multi-canal BPPV, *n* (%)				
TB	3 (1.5)	1 (0.9)	2 (2.2)	0.896^**^
MRC	3 (1.5)	1 (0.9)	2 (2.2)	0.597^**^
Posterior BPPV, total, *n* (%)				
TB	47 (23.4)	29 (26.9)	18 (19.4)	0.278
MRC	49 (24.4)	31 (28.7)	18 (19.4)	0.169
Lateral BPPV total, *n* (%)				
TB	16 (8.0)	11 (10.2)	5 (5.4)	0.320
MRC	21 (10.4)	14 (13.0)	7 (7.5)	0.305
Anterior BPPV total, *n* (%)				
TB	0 (0.0)	-	-	-
MRC	0 (0.0)	-	-	-
CAN BPPV, *n* (%)				
TB	52 (25.9)	31 (28.7)	21 (22.6)	0.408
MRC	56 (27.9)	34 (31.5)	22 (23.7)	0.282
CUP BPPV, *n* (%)				
TB	11 (5.5)	9 (8.3)	2 (2.2)	0.107^**^
MRC	16 (8.0)	14 (13.0)	2 (2.2)	0.010^**^

Please note that the proportion of males and the BPPV subtype of CUP was significantly higher in the population at the tertiary Dizziness Center. Please also note, that five patients had multicanal BPPV. Significant p-values are shown in italic. Multi-canal BPPV is defined as more than one affected SCC.

MRC, mechanical rotation chair. TB, traditional bedside. GP, general practitioner. CAN, canololithiasis. CUP, cupulolithiasis, SCC, semicircular canal.

^*^Missing data from 14 participants.

^**^ Compared using Fischers Exact test.

Following thorough diagnostic procedures, examinations with both test modalities rejected the BPPV diagnosis with 140 (69.7%) and 133 (66.2%) following TB- and MRC diagnostics, respectively. With 54 (26.9%) of the study participants, both test modalities agreed on a diagnosis of BPPV. With the remaining 21 (10.4%) study participants, a diagnosis of BPPV was placed in 14 out of 21 (66.6%) with an MRC, while TB diagnostics placed a diagnosis of BPPV in seven out of 21 (33.3%) (refer to Table 3). As a direct result hereof, we assumed that the MRC test modality was the most sensitive test modality. The MRC was therefore categorized as the “gold standard” test modality for BPPV diagnostics in the subsequent calculations of sensitivity and specificity.

**Table 3:** BPPV diagnostics overall with two separate test modalities.

MRC-diagnostics	TB-diagnostics		Total
	BPPV	No BPPV	
BPPV	54	14	68
No BPPV	7	126	133
Total	61	140	201

In 14 out of 21 study participants, where there was disagreement on the BPPV diagnosis, diagnostics with an MRC allowed the examiner to place a BPPV diagnosis.

MRC, mechanical rotation chair; TB, traditional bedside.

With placing a diagnosis of BPPV overall, agreement between the two test modalities was 89.6 [95% CI (84.5; 93.4)] and Cohen's kappa was 0.76 [95% CI (0.66; 0.86)]. Traditional bedside diagnostics was found to have a sensitivity of 79%, a specificity of 95%, a NPV of 90%, and a PPV of 89% in comparison with (gold standard) MRC diagnostics (refer to Table 4). If agreement included *all* BPPV specific characteristics (BPPV laterality, -location, and -subtype), agreement was found to be 83.6% [95% CI (77.7; 88.4)]. For detailed characteristics on lateral BPPV please refer to Table 5.

**Table 4:** Overall agreement, sensitivities, specificities, and predictive values with selected BPPV properties.

Condition	*n*	Agreement		TB- compared to MRC diagnostic			
		Percentage agreement	Cohens kappa	Sensitivity	Specificity	PPV	NPV
BPPV or no BPPV, overall	201	89.6 (84.5;93.4)	0.76 (0.66;0.86)	79.41 (67.88;88.26)	94.74 (89.46;97.86)	88.52 (77.78;95.26)	90.00 (83.79;94.42)
Posterior BPPV	201	90.0 (85.1;93.8)	0.73 (0.61;0.84)	77.55 (63.38;88.23)	94.08 (89.06;97.26)	80.85 (66.74;90.85)	92.86 (87.58;96.38)
Non–posterior BPPV	201	95.5 (91.7;97.9)	0.70 (0.52;0.89)	63.16 (38.36;83.71)	98.90 (96.09;99.87)	85.71 (57.19;98.22)	96.26 (92.44;98.48)
ENT Practice	93	86.0 (77.3;92.3)	0.62 (0.43;0.81)	69.57 (47.08;86.79)	91.43 (82.27;96.79)	72.73 (49.78;89.27)	90.14 (80.74;95.94)
Tertiary Dizziness Center	108	92.6 (85.9;96.7)	0.84 (0.74;0.95)	84.44 (70.54;93.51)	98.41 (91.47;99.96)	97.44 (86.52;99.94)	89.86 (80.21;95.82)
Sufficient cooperation	191	90.1 (84.9;93.9)	0.77 (0.67;0.87)	80.95 (69.09;89.75)	94.53 (89.06;97.77)	87.93 (76.70;95.01)	90.98 (84.77;95.25)
Impaired but acceptable cooperation	10	80.0 (44.4;97.5)	0.60 (0.15;1.00)	60.00 (14.66;94.73)	100.00 (47.82;100.00)	100.00 (29.24;100.00)	71.43 (29.04;96.33)
TB diagnostics first	101	90.1 (82.5;95.1)	0.79 (0.67;0.91)	85.37 (70.83;94.43)	93.33 (83.80;98.15)	89.74 (75.78;97.13)	90.32 (80.12;96.37)
MRC diagnostics first	100	89.0 (81.2;94.4)	0.70 (0.54;0.87)	70.37 (49.82;86.25)	95.89 (88.46;99.14)	86.36 (65.09;97.09)	89.74 (80.79;95.47)

Predictive values include both positive predictive values (PPVs) and negative predictive values (NPVs). Values are calculated separately with BPPV location (posterior/non-posterior), degree of cooperation (with TB diagnostics), site of inclusion (ENT Practice/tertiary Dizziness Center), and order of test modality (MRC/TB). Numbers are percentages and parenthesis describe confidence intervals (CI). MRC, Mechanical Rotation Chair. TB, Traditional Bedside. Please note that there is significantly lower PPV with the ENT Practice population compared to the tertiary Dizziness Center and that sensitivity decreases with impaired patient cooperation.

**Table 5:** Diagnostics of lateral BPPV with two different test positions.

BPPV location and -subtype	90-degree SRT position (*n* = 93)	60-degree SRT position (*n* = 108)	Total (*n* = 201)	*P*-value
Lateral BPPV overall, *n* (%)	7 (7.5)	14 (13.0)	21 (10.4)	0.305
Right lateral CAN BPPV, *n* (%)	3 (3.2)	4 (3.7)	7 (3.5)	1.000
Right lateral CUP BPPV, *n* (%)	1 (1.1)	6 (5.6)	7 (3.5)	0.126
Left lateral CAN BPPV, *n* (%)	3 (3.2)	4 (3.7)	7 (3.5)	1.000
Left lateral CUP BPPV, *n* (%)	1 (1.1)	4 (3.7)	5 (2.5)	0.376

Comparison of two different MRC test positions with the SRT: A 90-degree Supine Roll Test position (ENT Practice) and a 60-degree Supine Roll Test (tertiary Dizziness Center) with the use of a mechanical rotational chair. Please note that the 60-degree Supine Roll Test position resulted in higher numbers of lateral BPPV both on the left and right side and with both BPPV subtypes—especially pronounced with the BPPV subtype of cupulolithiasis. Please also note that five patients had bilateral lateral BPPV and that no differences were found to be statistically significant. Refer to Figures 2 and 3 for further details on the exact positioning.

CAN, canalolithiasis; CUP, cupulolithiasis; SRT, Supine Roll Test; MRC, Mechanical Rotation Chair.

The vast majority of participants (191 out of 201 (95.0%)) were able to cooperate sufficiently during TB diagnostics. With this part of the study population, an agreement of 90.1, a Cohens kappa of 0.77, a sensitivity of 81%, and a specificity of 95% was found. With the remaining ten participants with “impaired but acceptable cooperation,” an agreement of 80.0, a Cohens kappa of 0.60, a sensitivity of 60%, and a specificity of 100% was found (please refer to Table 4).

Diagnostics with exclusively posterior CAN is described in Table 6. Agreement on posterior BPPV was 90.0 [95% CI (85.1; 93.8)] with a Cohen's kappa of 0.73 [95% CI (0.61; 0.84)]. A diagnosis of non-posterior BPPV was placed in 16 (8.0%) and 21 (10.4%) of study participants with TB and MRC diagnostics, respectively. With placing a diagnosis of non-posterior BPPV, the agreement was 95.5 [95% CI (91.7; 97.9)] and Cohen's kappa was 0.70 [95% CI (0.52; 0.89)].

**Table 6:** BPPV diagnostics isolated to posterior canalolithiasis BPPV.

Traditional bedside diagnostics	Mechanical rotational chair diagnostics				Total
	No posterior CAN BPPV	Right posterior CAN BPPV	Left posterior CAN BPPV	Bilateral posterior CAN BPPV	
No posterior CAN BPPV	148 (94.9)	5 (16.7)	4 (28.6)	0 (0.0)	157 (78.1)
Right posterior CAN BPPV	1 (0.6)	23 (76.7)	0 (0.0)	0 (0.0)	24 (11.9)
Left posterior CAN BPPV	7 (4.5)	2 (6.7)	10 (71.4)	0 (0.0)	19 (9.5)
Bilateral posterior CAN BPPV	0 (0.0)	0 (0.0)	0 (0.0)	1 (0.0)	1 (0.5)
Total	156 (100.0)	30 (100.0)	14 (100.0)	1 (100.0)	201 (100.0)

Please note that in almost 95% of cases both diagnostic modalities agreed that the participants had no posterior CAN BPPV. With 8 participants, TB diagnostics placed a diagnosis of posterior CAN where the MRC diagnostics did not, and with 9 participants, MRC diagnostics placed a diagnosis of posterior CAN where TB diagnostics did not.

CAN, canalolithiasis; TB, traditional bedside; MRC, mechanical rotation chair.

With the two different versions of SRT, a diagnosis of lateral BPPV was placed in 21 out of 201 (10.4%). Of these 21 study participants, 14 (66.6%) were placed in the 60-degree supine position and 7 (33.3%) were placed in the 90-degree supine position (refer to Figures 2, 3 and Table 5).

## 4 Discussion

This study attempted to quantify the accuracy of positional tests in BPPV diagnostics by comparing TB diagnostics on an examination bed to diagnostics aided by an MRC in a population of elderly patients. So far, not many studies have been published on BPPV diagnostics with MRCs. To the best of the author's knowledge, three previous studies have delt with the diagnostic properties of MRCs compared to TB diagnostics. In accordance with those studies, our test results demonstrate that MRCs are more sensitive, and therefore must be considered more accurate, diagnostic tools for placing a BPPV diagnosis compared to TB diagnostics on an examination bed (7, 11, 21).

In accordance with the diagnostic criteria set forth by the Bárány Society and the AAO-HNS, all participants included in this study presented with a typical BPPV case history (4, 5). Also, a diagnosis of BPPV was only placed if both clearly defined *objective criteria* (BPPV characteristic positional nystagmus) as well as *subjective criteria* (concomitant vertigo with BPPV characteristic positional nystagmus) were met (5). At the moment, no golden standard BPPV diagnostic test modality exists. Instead, by applying strict and well-defined diagnostic criteria, we determined the most sensitive test modality as the test modality that detected the highest number of BPPV cases overall and classified this test modality as the superior diagnostic tool. Overall, we found MRC diagnostics to place a diagnosis of BPPV in the highest number of patients. Compared to MRC diagnostics, TB diagnostics had an overall sensitivity of 79 %, a specificity of 95 %, a PPV of 89 %, and a NPV of 90 %. The specificity and PPV are similar to previous study results with specificities of 91–98 % and PPVs of 88–97 %, and NPVs of 73–76 % (7, 11). However, both sensitivity (tertiary dizziness clinic) and NPV (both sites) with this study are higher compared to previous study findings with sensitivities of 70–71 % and NPVs of 73–76 % (7, 11). These findings substantiate that TB diagnostics should be considered a useful test modality when it comes to placing a BPPV diagnosis. However, with a sensitivity of 79% and a NPV of 90%, the examiner should be cautious in rejecting the BPPV diagnosis if patients present with a typical BPPV case history with negative positional testing following TB diagnostics. If the case history is highly suggestive of BPPV, then repetitive testing might increase the diagnostic accuracy. One study found that the average number of DH tests required to obtain a positive test result (posterior BPPV) was 1.15 with a range of one to three (22). Ultimately, if TB diagnostics remain negative, these patients should be referred to a highly specialized dizziness clinic for MRC testing. With specificities of 90+ percentages, however, a positive test result with TB diagnostics should be considered conclusive of BPPV, and bedside treatment maneuvers should then be initiated.

The most common BPPV subtype and -location is posterior CAN BPPV. This mantra has recently been challenged by an Indian study with approximately 4,000 patients, where almost equivalent percentages of posterior- and lateral canal BPPV was seen (23). Diagnostic tests included both the DH- and SRT tests, but in this study, the SRT tests preceded the DH-tests. As a direct consequence hereof, the order of diagnostic tests performed with this study was bilateral SRTs followed by bilateral DH tests, to prevent theoretical underdiagnosing of lateral canal BPPV. We were not, however, able to find a substantial or equal amount of lateral BPPV cases even though we changed the order of positional testing according to the study by Bhandari et al. mentioned above.

The two test modalities showed a near-perfect to substantial agreement (24) in diagnosing or rejecting BPPV, with an agreement rate of 89.6% and a Cohen's kappa of 0.76. Similar studies found an agreement of 0.81–0.83 and Cohen's kappa of 0.61–0.66 equivalent to a near-perfect to substantial agreement (7, 11). These studies were done with a similar study set-up and study methodology, but with a different type of MRC. With diagnostics of posterior canal BPPV alone, agreement of the two test modalities was 90% and Cohen's kappa was 0.73 equivalent to near-perfect to substantial agreement. Previous studies have found similar results with agreement between 0.83–0.89, and Cohen's kappa of 0.60–0.78 when comparing TB diagnostics and MRC diagnostics in detecting posterior CAN BPPV (7, 11). When considering diagnostics of non-posterior BPPV, previous studies have shown substantially lower agreement and Cohen's kappa values. This study also found substantially lower agreement and Cohen's kappa values with non-posterior BPPV diagnostics. This means that TB diagnostics does not necessarily provide unambiguous results with diagnostics of all other BPPV subtypes and -locations than posterior CAN BPPV.

One very important thing to consider with inter-study comparisons is the heterogeneity of different study populations. With BPPV, this is of paramount importance, because many parameters might differ, e.g. age, gender, laterality, subtype, location, number of SCCs being affected (single-, bilateral, and/or multi-canal), length of symptomatology, etiology, type of previous treatment(s), and number of previous treatment(s). Also, site specific parameters might influence both diagnostics and subsequent treatments offered (primary-, secondary- or tertiary site), level of expertise, local clinical instructions (order of tests (type and laterality), diagnostic criteria), type of equipment, degree/level of advanced equipment, referral pathways etc. Study characteristics are also often heterogeneous and therefore also not always directly comparable, e.g. prospective/retrospective, single- or multicenter. This multicenter study wanted to address this issue by involving two very different study sites: (1) a highly specialized tertiary University Hospital based dizziness clinic and (2) a primary/secondary ENT Practice. When comparing these two study populations, significant differences were seen between parameters like the male/female ratio, referral patterns (e.g. proportion of self-referrals, referrals from an ENT and other hospital departments), the proportion of patients diagnosed with BPPV overall and BPPV-specifics like primary BPPV, bilateral BPPV, mono canal BPPV as well as the CUP subtype of BPPV. The rather high number of significant differences between the two study populations clearly shows that BPPV populations are very heterogenous and therefore not always directly comparable.

The elderly are especially at risk of developing BPPV, as the incidence increases with age and peaks in individuals over 60 years of age (5). Moreover, dizziness and vestibular dysfunction affect one-third of adults aged 65 years and older and is linked to an increased risk of falls (25). However, not all patients with BPPV experience true vertigo. Especially elderly patients might frequently present with an atypical manifestation of BPPV, with primarily complaints of imbalance rather than vertigo (26, 27). Additionally, elderly patients may suffer from vestibular agnosia, that reduces the vestibular sensation of self-motion and causes either vague or completely absent sensations of dizziness or vertigo (9). Largely, this type of patients has not been included in this study, as all study participants presented with complaints of dizziness and/or vertigo. A Swiss study with a portable MRC, which tried to establish the prevalence of BPPV among retirement home residents who complained of dizziness or imbalance, found a point prevalence of 11.3% out of 16.6% complaining of dizziness (17). Also worth considering, when trying to compare TB diagnostics with MRC diagnostics among elderly is the degree of both involuntary cooperation (constraints of musculoskeletal system movements) and voluntary cooperation (constraints due to e.g. anxiety). Within a population of elderly, the examiners in this study only classified ten (5.0%) study participants to have “an impaired but acceptable degree of cooperation” in a population of elderly. This might suggest some kind of selection bias, because other similar studies on adults (all aged 18 or above) have found much higher proportions of patients not being able to fully cooperate during TB diagnostics. The two studies by Bech et al. (7) and Hentze et al. (11) found much higher, but very similar, proportions of participants that could not fully cooperate during TB diagnostics at 18.9% and 18.6%, respectively. Also, one very important thing to consider with TB diagnostics, is correct angling during the positional testing. A recently published study, that also compared TB diagnostics with MRC diagnostics (however, with a different MRC), found that correct angling during SRT (90- degree yaw axis turns to either side) was off target by 19.7–23.8 degrees (28). With SRT testing, this study used head turn and not entire body roll with TB diagnostics. This difference in methodology might, to some extent, explain why the MRC at both sites was superior in diagnosing lateral canal BPPV.

Because this study includes the use of a fairly new MRC, not many previous studies have been published. To the knowledge of the authors of this study, all previous studies have exclusively examined the use of this MRC in relation to BPPV treatment (16–18). Instead, this study looked at the diagnostic properties of placing a BPPV diagnosis with an MRC in comparison to TB diagnostics on an examination bed. Like similar studies, we also found that this MRC provides superior diagnostics in relation to especially non-posterior BPPV (7, 11). Currently, no consensus exists in terms of how to specifically perform the SRT with this MRC, so we decided to examine this parameter as well. Theoretically, one should perform the SRTs with the lateral SCCs completely vertical. However, this is not possible with this MRC at the moment. You can either position the patient completely in the supine position (90 degrees backward in the supine position) and then turn the patient 90 degrees to either side or position the patient 60 degrees backward and then turn the patient 90 degrees to either side. With either test method, the lateral SCCs will not be positioned in the vertical plane throughout the entire 90-degree rotation. At the private ENT site, examiners were able to diagnose lateral BPPV with 7 out of 93 (7.5%) and, at the tertiary dizziness center site, examiners were able to place a diagnosis of lateral BPPV with 14 out of 108 (13%). Overall, two thirds of the patients diagnosed with lateral BPPV underwent MRC diagnostics in the 60 degrees backward position. These numbers might indicate that the 60 degrees backward test position is superior to the 90-degree backward test position. However, differences were non-significant and the *per se* prevalence of lateral BPPV might also differ between the secondary- and tertiary study populations. In order to optimize the position of the lateral SCCs during SRTs, we recommend that a cushion, that forces the head 30-degrees forward, is positioned at the back of the head (like other similar MRCs). Also, future studies on this should include a test set-up with evaluation of the same patient that should undergo diagnostics with both versions of the SRT at a highly specialized (high lateral BPPV prevalence) site.

### 4.1 Strengths and limitations

This study was designed as a prospective, block-randomized crossover study to minimize bias. By choosing a prospective study design, the advantages include strong evidence for causality, a reduced risk of recall bias, a high data accuracy, an ability to measure multiple outcomes, and a minimization of selection bias. The same examiner performed all clinical examinations with the two different diagnostic modalities used with the same patient. By using the same examiner for both diagnostic procedures with the two different modalities, we ensured consistency with both the performance of the diagnostic procedures as well as the identification and interpretation of the objective findings. This was done to eliminate bias due to inter-examiner variation. All health care professionals, who contributed to the collection of data in relation to this study, were either already experienced clinicians or health care professionals who had undergone thorough training prior to initiation of this study. The number of health care professionals performing the diagnostic procedures in relation to this study was limited to two persons at each site. The same set of VNG-goggles (and accompanying software) with the vision denied option were used with both the TB diagnostic and the MRC diagnostics. This ensured that the eye monitoring properties were identical with both test modalities.

By adding different sites and types of sites (primary/secondary and tertiary), the population heterogeneity increases. This might be seen as both a strength and a limitation. By including a more diverse BPPV population, the generalizability of study results increases. However, direct inter-study comparisons might prove more difficult with this type of population. Inclusion of exclusively elderly patients above the age of sixty might also be considered both a strength and a limitation as this is a highly selected group of patients with specific characteristics. On one side, it makes the study results suitable for recommendations for a selected group of patients, but on the other side, it makes study results less generalizable.

A clear limitation of this study methodology includes the lack of examiner blinding. In a clinical context, this means that the interpretation of the second diagnostic modality might, unintentionally, have been influenced by the objective findings and subjective symptoms observed with the first diagnostic modality. This might have applied a confirmation bias to the study results. In this case, the performance of the same diagnostic modality should increase if it was used as the second diagnostic modality. Another potential limitation of this study is the risk of a carryover effect. This might have occurred in relation to repetitive positional testing. Already with the initial positional testing with the first diagnostic modality, the conditions for the following positional tests might have changed. So, the order of testing might influence the test conditions because BPPV is a dynamic disease entity. To reduce the risk of this carryover effect, the order of diagnostic modality was randomized, and a period of at least 15 min was included between the use of the two diagnostic modalities to avoid issues with fatiguability of positional nystagmus.

Rather strict diagnostic criteria were used in this study. Because “a typical BPPV case history” was considered a prerequisite, patients with vestibular agnosia supposedly have not been included in this study population and therefore a selection bias might have been introduced. If the inclusion criteria (and diagnostic criteria) had been less strict, then a wider group of elderly patients might have been included (8, 9, 17).

### 4.2 Clinical implication of study results

Based on the abovementioned study results, as well as other similar study results, TB diagnostics can be considered a useful first line tool for BPPV diagnostics overall and especially with diagnostics of posterior CAN BPPV (7, 11). One must therefore be cautious when ruling out BPPV in a patient who presents with a typical BPPV case history who has negative positional tests with TB diagnostics. Based on the current evidence, TB diagnostics should be performed whenever BPPV is suspected. If this confirms that the patient has posterior CAN BPPV, then traditional repositioning maneuver(s) should be performed (5, 29). If traditional positional diagnostics prove negative or if other BPPV subtypes and/or -locations are diagnosed, then the patient should be referred for MRC diagnostics and subsequent highly specialized and targeted treatment(s) which have proven to be very successful (30).

This study also adds to the existing pool of evidence in relation to MRC diagnostics – especially in relation to this rather new MRC.

## 5 Conclusion

Overall agreement between the two test modalities was substantial to near-perfect. However, with MRC diagnostics, a diagnosis of BPPV can be placed more frequently and more accurately than with TB BPPV diagnostics. Especially diagnostics of non-posterior BPPV seem superior with the use of an MRC, as the sensitivity with TB diagnostics is only 63%. With TB diagnostics, diagnostic capabilities decrease with impaired participant cooperation.

Traditional bedside diagnostics on an examination bed is still recommended as a first line diagnostic tool for BPPV. With placing a BPPV diagnosis, an MRC may be considered if (1) the BPPV diagnosis is ambiguous (especially in patients with a typical BPPV case history where TB diagnostics are negative), (2) TB diagnostics suggest non-posterior BPPV, (3) patient cooperation is low with TB diagnostics. Also, with this type of MRC, SRTs should be performed with the patient positioned 60 degrees backward for optimal diagnostics of lateral canal BPPV.

## Data Availability

The raw data supporting the conclusions of this article will be made available by the authors, without undue reservation.
